# *Cereus sinensis* Polysaccharide Alleviates Antibiotic-Associated Diarrhea Based on Modulating the Gut Microbiota in C57BL/6 Mice

**DOI:** 10.3389/fnut.2021.751992

**Published:** 2021-12-13

**Authors:** Mingxiao Cui, Yu Wang, Jeevithan Elango, Junwen Wu, Kehai Liu, Yinzhe Jin

**Affiliations:** ^1^Department of Biopharmaceutics, College of Food Science and Technology, Shanghai Ocean University, Shanghai, China; ^2^National R&D Branch Center for Freshwater Aquatic Products Processing Technology (Shanghai), Shanghai, China

**Keywords:** *Cereus sinensis*, polysaccharide, antibiotic-associated diarrhea, gut microbiota, short chain fatty acid

## Abstract

The present study investigated whether the purified polysaccharide from *Cereus sinensis* (CSP-1) had beneficial effects on mice with antibiotic-associated diarrhea (AAD). The effects of CSP-1 on gut microbiota were evaluated by 16S rRNA high-throughput sequencing. Results showed that CSP-1 increased the diversity and richness of gut microbiota. CSP-1 enriched *Phasecolarctobacterium, Bifidobacterium* and reduced the abundance of *Parabacteroides, Sutterella, Coprobacillus* to near normal levels, modifying the gut microbial community. Microbial metabolites were further analyzed by gas chromatography-mass spectrometry (GC-MS). Results indicated CSP-1 promoted the production of various short-chain fatty acids (SCFAs) and significantly improved intestinal microflora dysfunction in AAD mice. In addition, enzyme linked immunosorbent assay and hematoxylin-eosin staining were used to assess the effects of CSP-1 on cytokine levels and intestinal tissue in AAD mice. Results demonstrated that CSP-1 inhibited the secretion of interleukin-2 (IL-2), interleukin-1β (IL-1β) and tumor necrosis factor-α (TNF-α) and improved the intestinal barrier. Correspondingly, the daily records also showed that CSP-1 promoted recovery of diarrhea status score, water intake and body weight in mice with AAD. In short, CSP-1 helped alleviate AAD by regulating the inflammatory cytokines, altering the composition and richness of intestinal flora, promoting the production of SCFAs, improving the intestinal barrier as well as reversing the dysregulated microbiota function.

## Introduction

Antibiotics are highly effective in treating numerous bacterial or pathogenic infections (1). Nevertheless, the misuse or inappropriate use of antibiotics may alter the structure of the gut microbiota, disrupt the microbial balance, and thereby cause potential clinical complications in the host (2). As a common intestinal complication due to the use of antibiotics, AAD may manifest as symptoms, such as mild diarrhea, colitis and toxic megacolon. Many reports have shown that long-term probiotic therapy can significantly improve intestinal flora, promote the recovery of intestinal tissue architecture, and alleviate systemic inflammation, suggesting that probiotics are beneficial to the recovery of AAD mice (3). At present, probiotic therapy is the principal method to alleviate AAD disease. However, probiotics were easily inactivated. Therefore, it was still a hot topic to find effective alternatives with long-term stable storage.

Prebiotics have also been reported to have a good effect on restoring intestinal balance and have a long storage period (4). Some natural polysaccharides with few adverse effects and particular biological activities are important prebiotics (5, 6). It has also been confirmed that certain polysaccharides have shown efficacy in alleviating or treating certain diseases, involving colitis, diabetes and AAD, by upregulating the healthful bacteria and suppressing the maleficent bacteria to regulate the intestinal flora (7, 8). For instance, polysaccharides derived from inulin and yam were reported to up-regulate the abundance of bacteria producing lactic acid and SCFAs, reduce the abundance of *Bacteroides, Proteobacteria* and sulfate-reducing bacteria, modulate the gut microbiota composition and function and ultimately ameliorate colitis of rats (9). Furthermore, polysaccharides isolated from *Schisandra chinensis* reduced intestinal mucosal damage based on beneficial regulation of intestinal flora, thereby helping to alleviate AAD (10). Therefore, certain natural polysaccharides may be potential prebiotic agents to effectively alleviate AAD disease based on their beneficial effects on the intestinal flora.

*Cereus sinensis* belonging to Actiniaria, *HormathiidaeCarlgren, CalliactisVerrill* grown mainly along the Pacific coast (11). At present, many substances with toxic or important functions, such as anti-cancer, antibacterial, anticoagulant, and anti-inflammatory, have been found and extracted from sea anemones (12–14). Moreover, the research on sea anemone mainly focused on the field of proteins, polypeptides, the toxins structure and biological activities (15–18). Nevertheless, there were few reports about sea anemone polysaccharide. In our previous research, a novel *Cereus sinensis* polysaccharide (CSP-1) was obtained and further analyzed (19). Its monosaccharide composition mainly includes Fucose, Mannose, and Glucose (14.9: 1.2: 1.0), and its mean molecular weight is 56,335 Da. The glycosidic linkage of CSP-1 was inferred as 1 → 2, 1 → 4, 1 → 2, 6, and 1 → 4, 6. The beneficial effects of CSP-1 on the body are still unclear, which also limits its development and utilization. Currently, probiotics are commonly used to treat antibiotic-associated diarrhea. However, they are easily inactivated in the gastrointestinal environment and require high storage conditions. As a prebiotic, polysaccharides have good stability in the gastrointestinal environment and room temperature, and may also positively regulate the intestinal microbiota and have beneficial effects on intestinal diseases. Thus, we decided to evaluate the unexplored polysaccharide CSP-1's regulatory effects on the disordered intestinal flora and its beneficial effects on AAD, providing a candidate effective ingredient for the treatment of AAD. This could provide a theoretical basis for CSP-1 as a stable and effective prebiotic for AAD therapy, and at the same time provide the applied basis for the development of CSP-1 as a functional food ingredient or additive to alleviate AAD. AAD has been reported to be closely bound up with changes in the structure and function of intestinal flora, physiological performance, intestinal barrier, serum cytokine secretion and SCFAs production in mice (20). Based on the above indicators, the beneficial effects of CSP-1 were further explored.

## Materials and Methods

### Samples and Materials

Lincomycin hydrochloride (LH) was purchased from Anhui Shuanghe Pharmaceutical Co., Ltd. (Anhui, China). ELISA kits were purchased from Shanghai MLBIO Biotechnology Co., Ltd. QIAamp DNA stool mini kit was manufactured by TIANGEN (Germany). Other reagents were purchased from Sinopharm Chemical Reagent Co., Ltd. (Shanghai, China).

According to our previous methods, CSP-1 was isolated from *Cereus sinensis* (19, 21). After being soaked in NaCl solution (2%) to remove impurities, fresh *Cereus sinensis* was crushed and mixed with an equal volume of acetone for 12 h. The solution was filtered and freeze-dried to obtain degreased *Cereus sinensis* powder. The powder was mixed with distilled water at 72°C for 3 h and then precipitated with 3 times the volume of ethanol for 2 days. The above solution was centrifuged to obtain the precipitate, which was the crude polysaccharides. 50 mg/mL crude polysaccharide solution was mixed with 3% trichloroacetic acid for 12 h to remove protein. The solution was centrifuged, concentrated, dialyzed (3500D MWCO), and lyophilized. 5 mL of 20 mg/mL polysaccharide solution were further purified by column chromatography, involving DEAE-52 ion-exchange column and Sephadex G-100 column. 0.3 mol/L NaCl solution was used as eluate. The solution containing polysaccharides was collected, concentrated, dialyzed, and lyophilized to obtain CSP-1.

### Ultraviolet (UV) and Fourier Transform Infrared (FT-IR) Spectral Analysis of CSP-1

A UV-752 spectrophotometer (Qingdao Lubo Jianye Environmental Protection Technology Co., Ltd., Qingdao, China) was used to record the UV spectra of CSP-1 (scanning range 200–400 nm). FT-IR spectrometer (Tianjin Gangdong Technology Co., Ltd., Tianjin, China) was used to measure the FT-IR spectra of CSP-1 (scanning range 4,000–400 cm-1). The polysaccharide sample was mixed with dried KBr and then pressed into a thick pellet for FT-IR determination. The FT-IR and UV spectra of CSP-1 were shown in Supplementary Figure S1.

### Animals and Experimental Design

All experimental procedures were approved by the Committee for Animal Research of Shanghai Ocean University, China (SCXK (HU) 2007-0003). At the same time, animal experiments were carried out strictly following the Guidelines for the Care and Use of Laboratory Animals. Efforts were made to maximize the well-being of mice and minimize their suffering.

**Figure 1:**
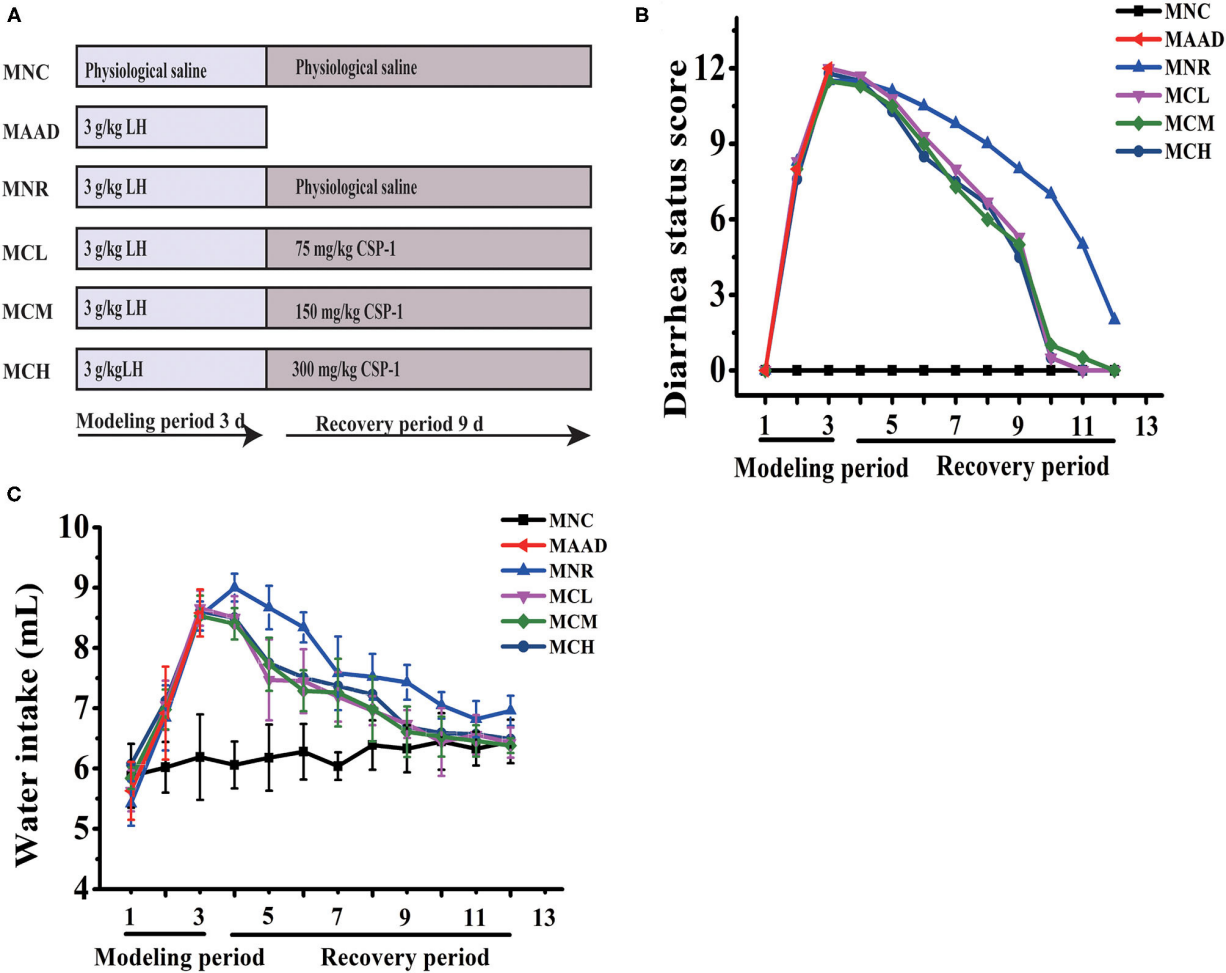
Schematic diagram in the experiment **(A)**; Changes of diarrhea status score and water intake **(B,C)**. Values are shown as means ± SD.

C57BL/6 male mice, 6–8 weeks, were obtained from the Shanghai Jiesijie Experimental Animal Co., Ltd and raised in a standard environment (12 h light-dark cycle, 22 ± 0.5°C, RH: 50 ± 5%) for 1 week. And then mice were randomly distributed into 6 groups with 3 mice per group. It was reported that short-term high-dose of LH will cause diarrhea in mice, which is mainly due to the disturbance of intestinal flora and inflammation of the intestinal tissues caused by LH (22). Therefore, LH is often used in the establishment of AAD models. Mice in the antibiotic-associated diarrhea group (MAAD) were gavaged with LH for only 3 days. Mice in the natural recovery group (MNR) were gavaged with LH for 3 days and then gavaged with physiological saline for the next 9 days. Mice in the Low- (MCL), medium- (MCM), and high- (MCH) dosage CSP-1 groups were gavaged with LH for 3 days and then gavaged with CSP-1 (MCL, MCM and MCH groups were 75, 150 and 300 mg/kg, respectively) for the next 9 days. Mice in the normal control group (MNC) were gavaged with physiological saline for 12 days. The dosage of LH in this experiment was 3 g/kg. Mice were gavaged twice a day during the experimental period. The experimental design was shown in Figure 1A.

The state of all mice was recorded daily, such as body weight, water intake, and diarrhea status. Diarrhea status was evaluated as previously reported (Table 1) (20). At the end of the experiment, mice were euthanized. The caecal specimens were temporarily immersed in formalin solution (10%) for histological observation. The caecal contents were collected and next stored at −80°C.

**Table 1:** Diarrhea status scoring methods.

**Scores**	**Diarrhea status**
0	Normal mental state and stools
1	General mental state, loose and nonstic perianal stools
2	Bad mental state, adhesion stool at anus, inappetence, weight reduction

### Histological Examination

After overnight fixation, the samples of each group were embedded, sectioned and stained by Shanghai Viao Biotechnology Co., Ltd. Briefly, caecal specimens were prepared by ethanol dehydrating and paraffin embedding (23). The sliced (thickness of 5 μm) specimens were stained with hematoxylin and eosin. The sections were observed under a light microscope (40 ×) and images were obtained.

### Determination of Serum Cytokine Secretion and SCFAs Production

The blood was collected and then centrifuged at 2,000 rpm for 10 min. The serum was obtained by collecting the supernatants. The secretion of TNF-α, IL-1β, and IL-2 was measured using ELISA kits according to the corresponding instructions.

Pretreatment of caecal content was carried out as previously reported (24). 100 mg cecal contents were mixed with 0.4 mL of distilled water and centrifuged at 5,000 rpm for 20 min. The supernatant was mixed with 50% H_2_SO_4_ (0.2 mL) and 50 μg mL^−1^ (1 mL) solution containing diethyl ether and 2-methylvaleric acid. The above solution was centrifuged at 12,000 rpm for 10 min and then placed at 4°C for 30 min. The upper liquid was used for the determination of SCFAs. Furthermore, standard solutions of SCFAs (acetate, propionate, isobutyrate, butyrate, 2-methylbutyrate, valerate and hexanoate) at different concentrations (5 μg mL^−1^, 10 μg mL^−1^, 50 μg mL^−1^,100 μg mL^−1^, 200 μg mL^−1^, 400 μg mL^−1^, 1,000 μg mL^−1^) were prepared in ether. 2-methylpentanoic acid was mixed with diethyl ether as an internal standard solution. The SCFAs were detected using Agilent 7890A-5975C GC-MS System (Agilent, USA). The conditions were as follows: injection volume: 1 μL; carrier gas helium at a flow rate of 1 mL/min; the split ratio was 5: 1; injector temperature: 250°C. column temperature program: 100°C (maintained for 5 min) to 160 °C at 5°C/min; next increased to 240°C (maintained for 10 min) at 40°C/min.

### 16S rRNA High-Throughput Sequencing and Bioinformatics Analysis

DNA from the intestinal flora was obtained from the cecal contents using the DNA stool kit. PCR amplification of the V3-V4 region of bacterial 16S rRNA gene was carried out using forward primer (5′-ACTCCTACGGGAGGCAGCA-3′) and reverse primer (5′-GGACTACHVGGGTWTCTAAT-3′). Data can be obtained at NCBI with accession no. SUB9203775. The next procedures were the collection, purification, fluorescence quantification of amplification products and preparation of sequencing library. Sequencing of PCR products was performed by the Illumina MiSeq (Illumina, United States) sequencing platform at Personal Biotechnology Co., Ltd (Shanghai, China). The paired-end sequence was filtered using the sliding window method, then paired and connected using the FLASH software (v1.2.7), identified and further assigned to the corresponding sample to obtain the valid sequence for each sample (25). USEARCH (v5.2.236) was applied to filter the sequences obtained above and to cluster the superior reads into operational taxonomic units with a 97% similarity threshold. Using QIIME (v1.8.0) (26). Gut microbiota was analyzed by Illumina MiSeq sequencing based on QIIME analysis (27).

### Statistical Analysis

The data was presented as mean ± standard deviation (SD). A significant difference between groups was performed by one-way analysis of variance (ANOVA) followed by Duncan's multiple range tests using SPSS statistics 17.0 (IBM, USA). Differences were considered significant at *P* < 0.05.

## Results

### Effects of CSP-1 on Diarrhea Status Scores, Water Intake, Bodyweight

During LH intragastric administration, the mice showed a gradual increase in diarrhea status score and drinking water (Figures 1B,C). After 3 days, these mice exhibited 100% diarrhea. From the 4th day, the diarrhea status scores and water consumption of the MCL, MCM and MCH groups began to drop. As for the MNR group, the diarrhea status scores began to drop from the 4th day, while water intake began to decrease from the 5th day. On the 12th day, in terms of diarrhea status score and water consumption, the MNR group was obviously higher than the MNC group, while the MCL, MCM and MCH groups were similar to the MNC group. The bodyweight of the mice treated with the LH significantly decreased (Table 2). During the recovery period, the bodyweight of each group was gradually increasing. At the end of the experiments, the bodyweight of the mice in the MCL, MCM and MCH groups was higher than their starting weight, while the bodyweight of the mice in the MNR group was still lower than their starting weight.

**Table 2:** Effects of CSP-1 on the body weight of mice.

**Groups**	**Bodyweight (g)**		
	**Day 0**	**Day 3**	**Day 12**
MNC	23.65 ± 0.47	23.80 ± 0.46	24.81 ± 0.60
MAAD	23.96 ± 0.37	19.83 ± 0.60	-
MNR	23.45 ± 0.53	20.23 ± 1.13	22.94 ± 0.71
MCL	23.90 ± 0.76	20.39 ± 0.67	24.73 ± 0.69
MCM	23.14 ± 0.72	19.55 ± 0.82	23.74 ± 0.65
MCH	24.07 ± 0.74	21.53 ± 0.75	24.24 ± 0.51

*Values are shown as means ± SD*.

### Effects of CSP-1 on Histological Changes

**Figure 2:**
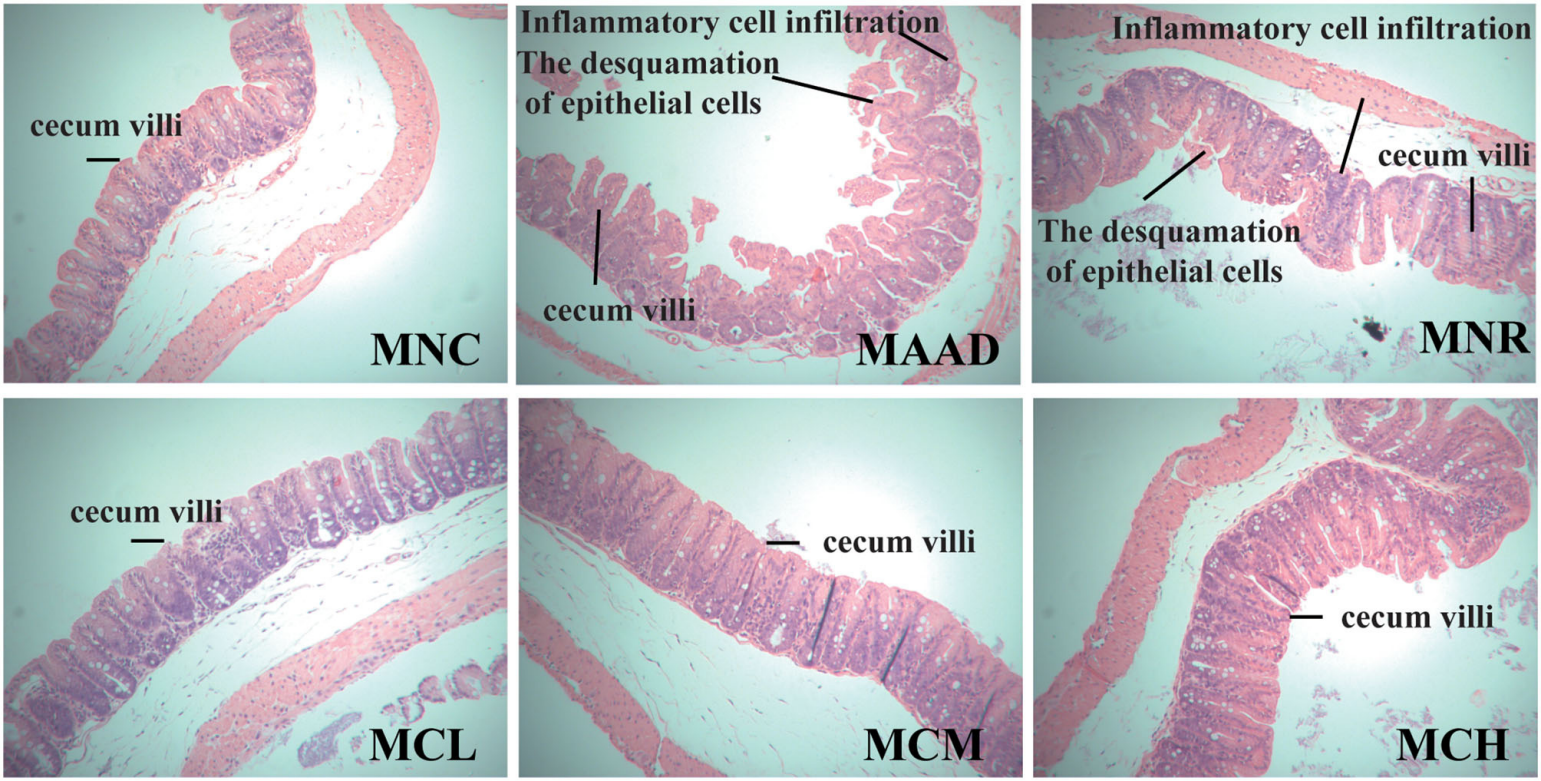
Histological changes of the cecum. Values are shown as means ± SD.

The cecal tissue in MAAD group exhibited serious histopathological changes, including edema, massive inflammatory cell infiltration, as well as villus shortening, sparse and irregular arrangement (Figure 2). Compared with the MNR group, CSP-1 significantly reduced the inflammatory cell infiltration and edema of the cecal tissue. Moreover, the cecal villi of the MCL, MCM and MCH groups were longer, thinner and relatively regular arrangement. There were no obvious differences among the MCL, MCM and MCH groups.

### Effects of CSP-1 on Inflammatory Cytokines Production

**Figure 3:**
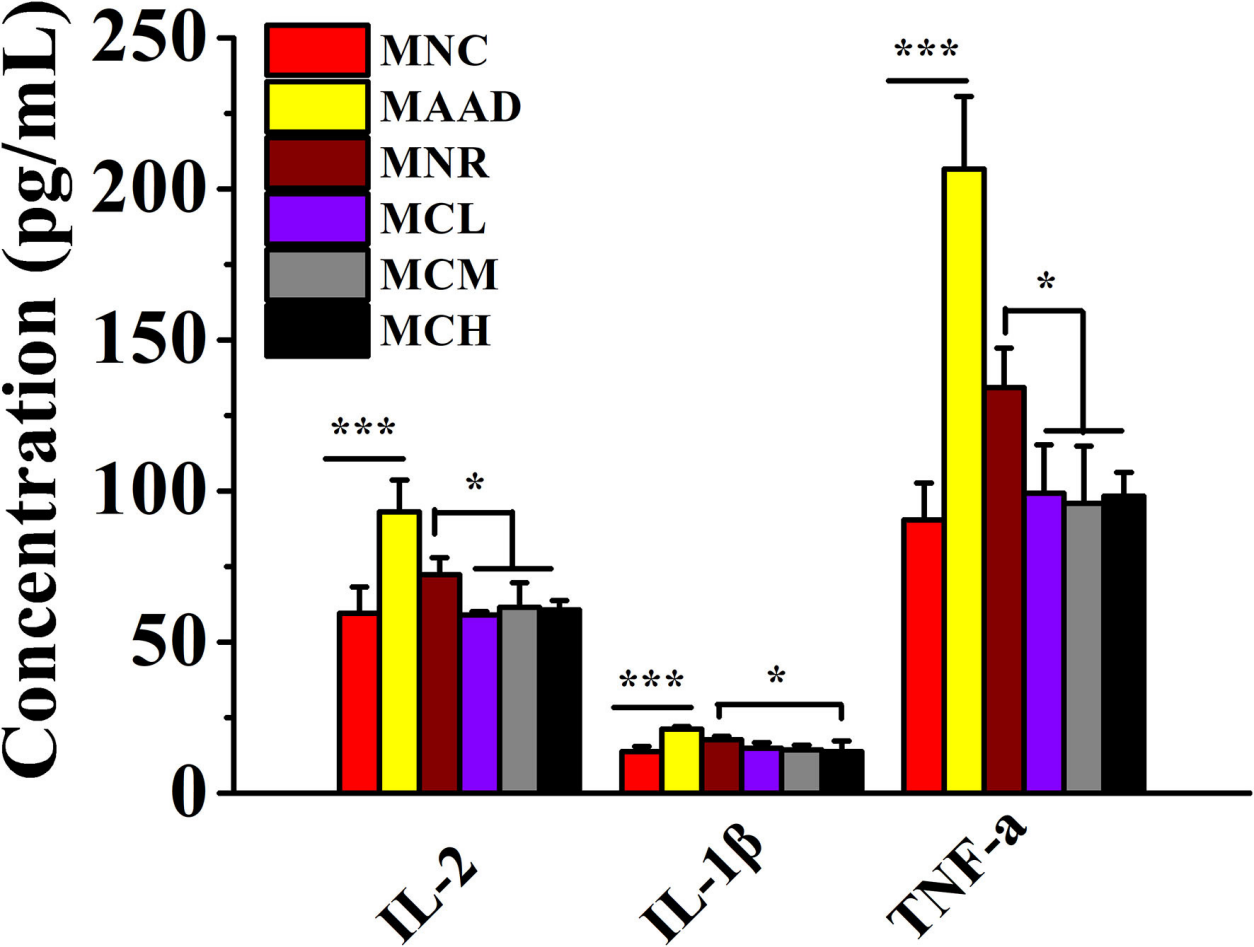
Changes in the secretion of inflammatory factors. Values are shown as means ± SD. *p < 0.05, ***p < 0.001.

LH treatment significantly stimulated the secretion of IL-2, IL-1β, and TNF-α in the serum of mice (Figure 3). Low, medium and high doses of CSP-1 significantly down-regulated levels of IL-2, TNF-α and high dosage of CSP-1 significantly decreased the level of IL-1β compared with the MNR group. No significant differences were observed among the MCL, MCM MCH, and MNC groups in the secretion of IL-2, IL-1β and TNF-α.

### Effects of CSP-1 on SCFAs Production

**Figure 4:**
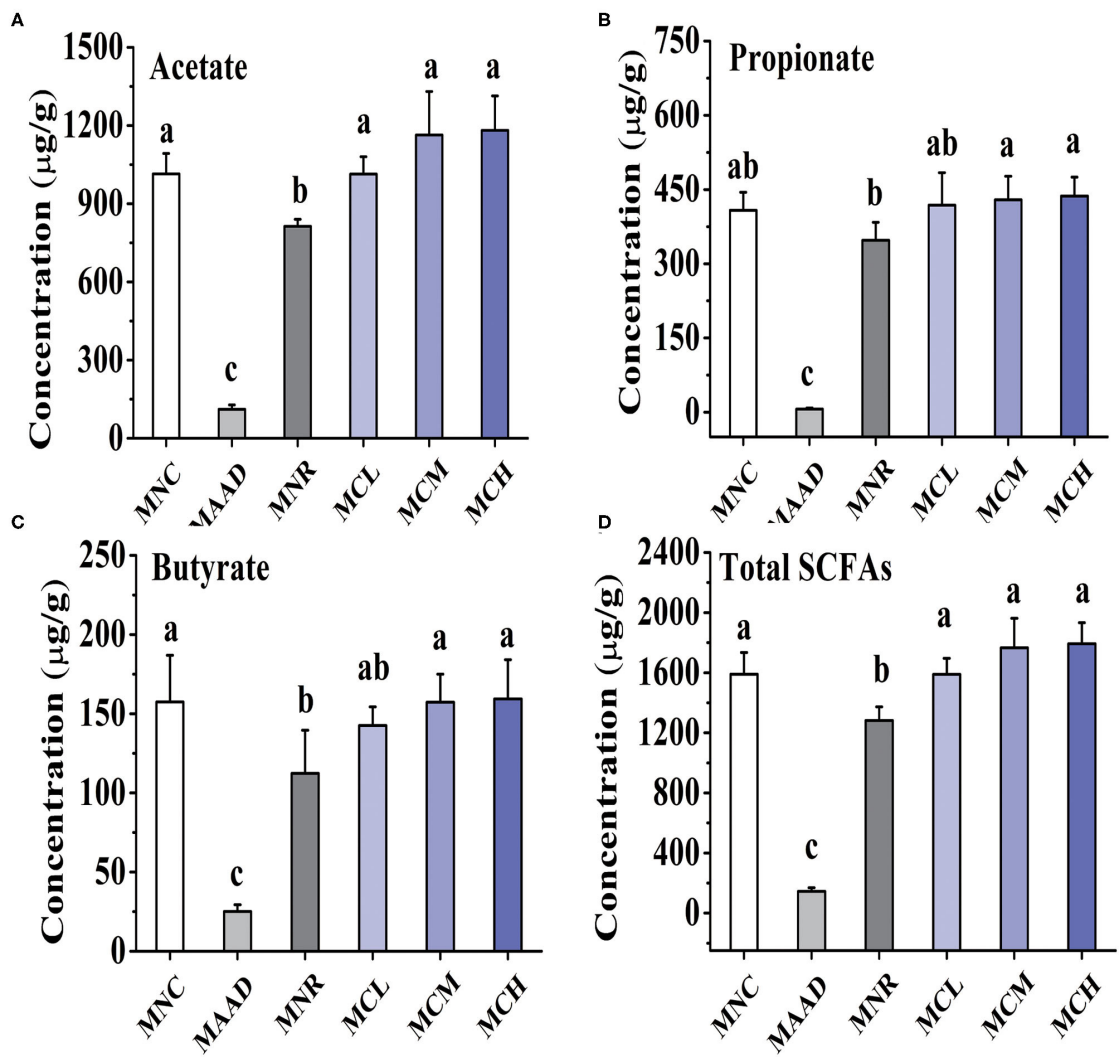
Changes in concentrations of acetate **(A)**, propionate **(B)**, butyrate **(C)** and total SCFAs **(D)**. Values are shown as means ± SD. Different letters are significant differences (*p* < 0.05).

Mice treated with low, medium and high dosage CSP-1 showed significant enhancements in acetate and total SCFAs production, compared with mice of the MNR group (Figure 4). Furthermore, mice treated with medium and high dosage CSP-1 also showed significant enhancements in propionate and butyrate production. CSP-1 treatment and MNC groups had no significant difference in terms of the production of acetate, propionate, butyrate and total SCFAs.

### Effects of CSP-1 on the Gut Microbiota

**Figure 5:**
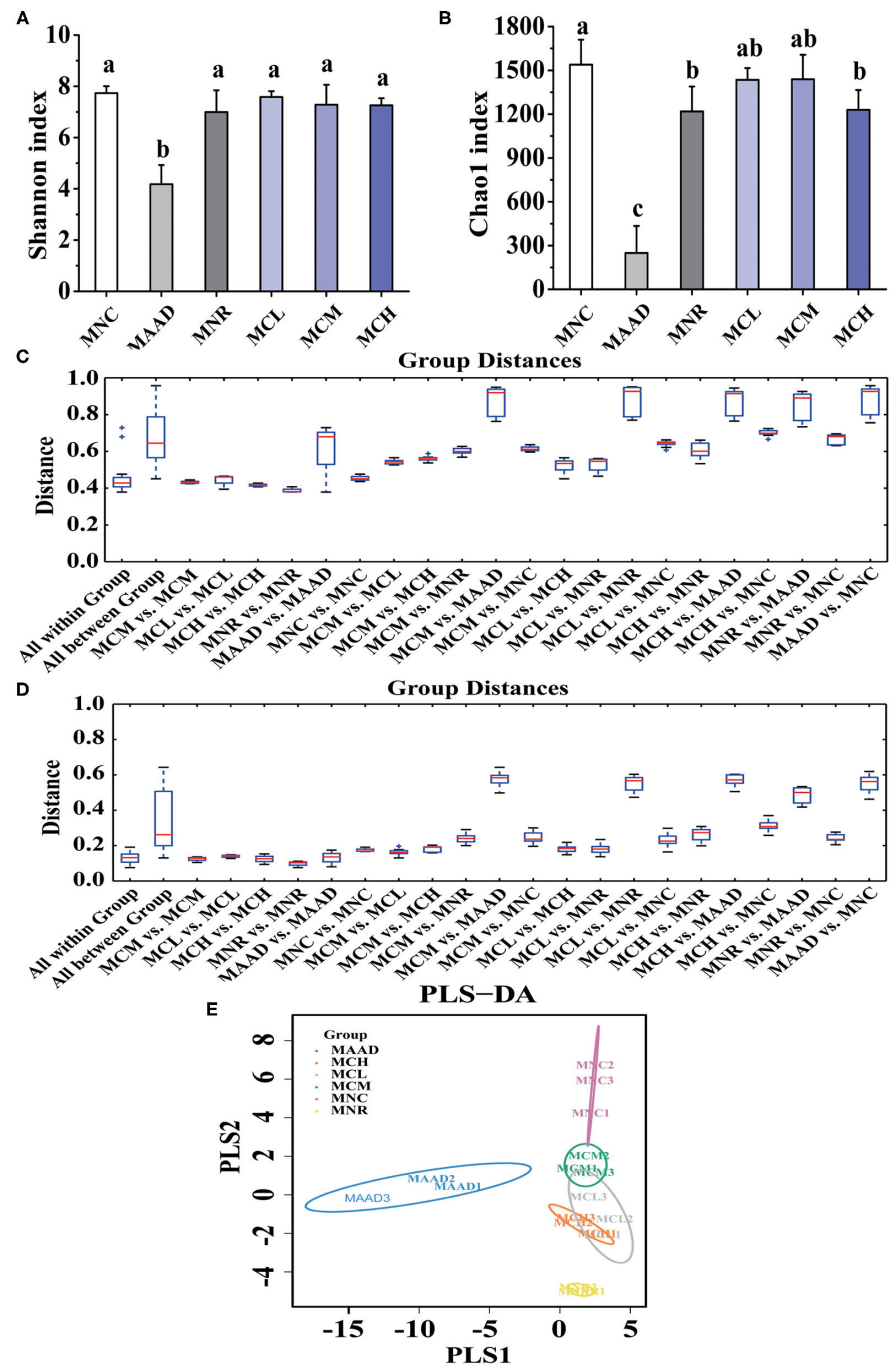
α diversity analysis, **(A,B)** Shannon and Chao 1 indices, and β diversity analysis, **(C,D)** Unweighted and Weighted UniFrac-distance box-line graph, **(E)** PLS-DA analysis, of the gut microbiota. Values are shown as means ± standard deviation (SD). Different letters are significant differences (*p* < 0.05).

Shannon and Chao 1 indexes were used to evaluate the diversity and richness of intestinal flora, respectively (Figures 5A,B). In terms of Shannon and Chao 1 indices, MNR, MCL, MCM and MCH groups were significantly higher than the MAAD group, while MCL, MCM and MCH groups were closer to the MNC group compared to the MNR group. In terms of Chao 1 index, MNC group was significantly higher than MNR group, while there was no significant difference among MCL, MCM and MNC group.

β diversity analysis (Figures 5C,D) showed that the MCL and MCM groups were closer to the MNC group than the MNR group. Meanwhile, the MNR group is closer to the MAAD group than the MCL, MCM, and MCH groups. PLS-DA analysis also demonstrated the structural changes of intestinal flora among the groups (Figure 5E). The significant separation between the MAAD and MNC groups indicated the successful establishment of the AAD model. Figure 5E also revealed that the MCL, MCM and MCH groups were closer to the MNC group than the MNR group.

**Figure 6:**
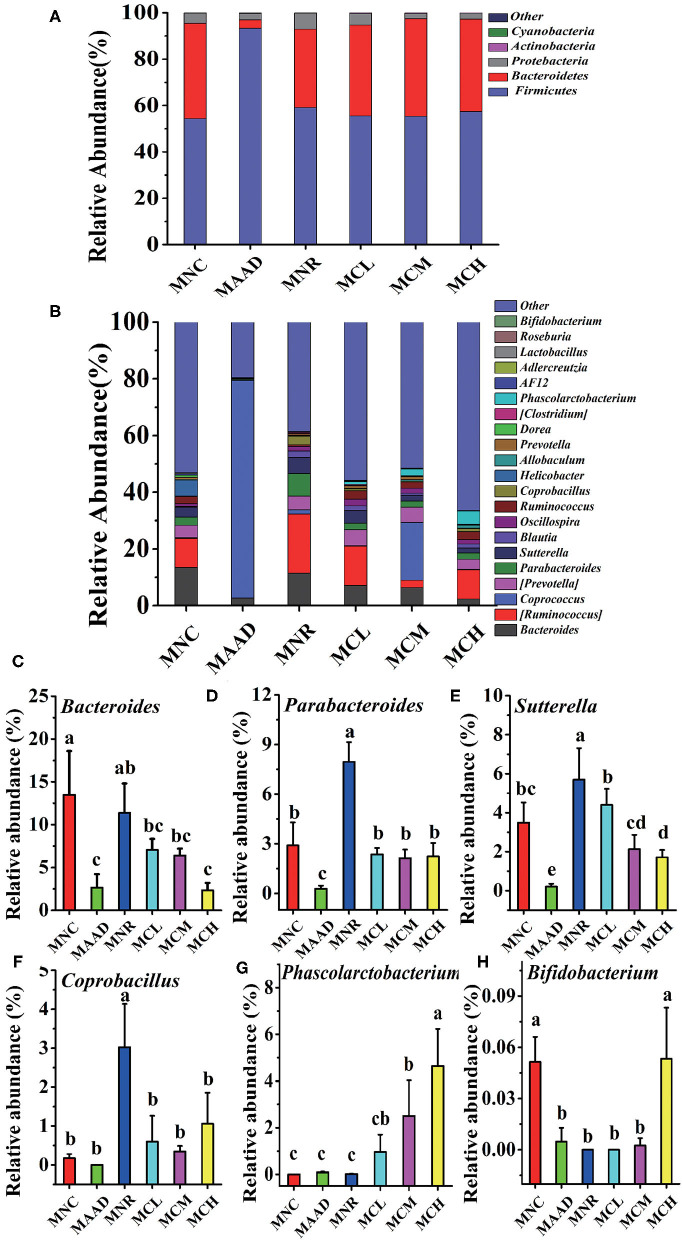
Changes of microbial communities at different levels. **(A,B)** Phylum and Genus levels. **(C–H)** Comparison of intestinal flora with major differences at the genus level. Values are shown as means ± SD.

The main components of each group included *Firmicutes, Bacteroidetes, Proteobacteria, Actinobacteria* at the phylum level (Figure 6A). However, their abundance was different. The MAAD group had significantly higher *Firmicutes* and lower *Bacteroidetes* and *Proteobacteria* than the MNC group. Compared with the MNR group, *Bacteroides* was enriched, while *Proteobacteria* and *Firmicutes* were down-regulated in the MCL, MCM and MCH groups. MNC, MCL, MCM and MCH groups in *Firmicutes* had no significant difference. Figure 6B showed the genera with the highest abundance in the MAAD group were *Coprococcus*. Compared to the MAAD group, CSP-1 significantly reduced *Coprococcus* and increased other bacterial communities in the MCL, MCM and MCH groups. Compared with MNR group, MCL, MCM and MCH groups had lower abundance of *Bacteroides, Parabacteroides, Sutterella, Coprobacillus* and higher abundance of *Phasecolarctobacterium* and *Bifidobacterium* (Figures 6C–H).

### Prediction of Metabolic Function of the Gut Communities

**Figure 7:**
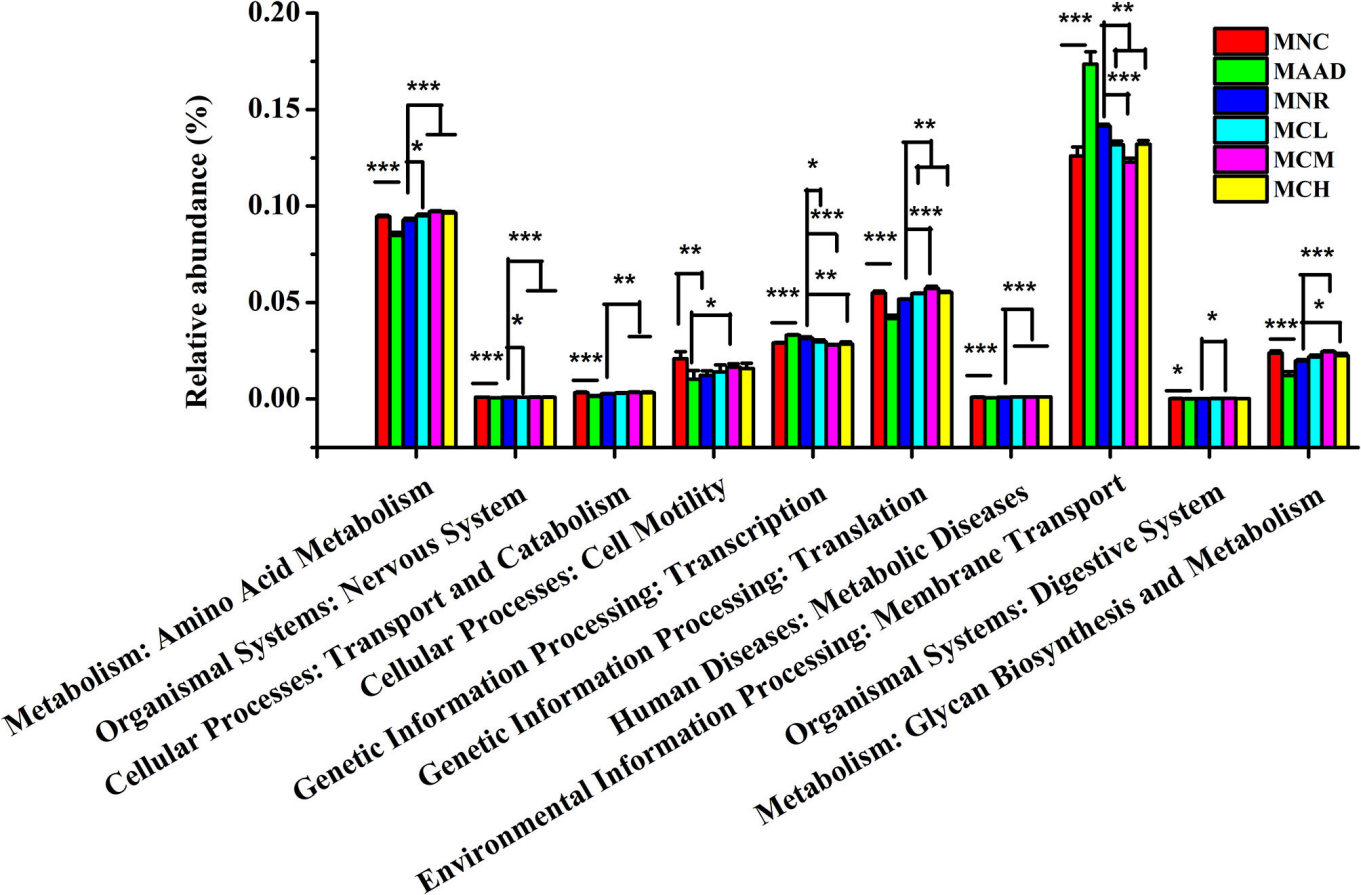
Prediction of metabolic function by PICRUSt analysis. Values are shown as means ± SD. Different letters are significant differences. *p < 0.05, **p < 0.01, ***p < 0.001.

LH disrupted normal microbial metabolic pathways in the gut community of mice, like mice in the AAD group (Figure 7). Compared to the MNR group, amino acid metabolism, nervous system, translation, metabolic diseases were obviously enhanced and transcription, membrane transport was significantly weakened in three CSP-1 treated groups. At the same time, mice treated with a medium and high dosage of CSP-1 showed significant enhancements in transport and catabolism, glycan biosynthesis and metabolism. In addition, mice treated with a medium dosage of CSP-1 also showed significant enhancement in digestive system. Moreover, three CSP-1 treated groups and the MNC group did not show significant differences in transport and catabolism, transcription, membrane transport, digestive system, glycan biosynthesis and metabolism. Furthermore, CSP-1 also had good effects on regulating amino acid metabolism, cell motility and translation.

## Discussions and Conclusions

AAD was closely connected with gut microbiota dysbiosis, intestinal structural changes, inflammation, SCFAs production (28). Compared to normal mice, mice with AAD manifested an imbalanced gut microbial environment, increased serum inflammatory cytokine levels, decreased SCFAs production, a destroyed gut structure. These were consistent with mice of the MAAD group. At present, it has been reported that natural polysaccharides as prebiotics helped the resistance of AAD by modulating the intestinal flora. For instance, a polysaccharide from Chinese yam alleviated AAD disease by upregulating the abundance of probiotics, suppressing the growth of potential pathogen, repairing the intestinal microbiota disorder, and up-regulating the concentration of SCFAs (9). These provided a reliable basis for natural polysaccharides as potential prebiotic agents to play a beneficial role by regulating intestinal flora.

Gut microbiota was closely associated with inflammation, gut mucosal dysfunction, SCFAs and certain diseases such as AAD and colitis. It is reported that certain gut bacteria and their metabolites can activate the mucosal immune system, which leads to inflammation and gut mucosal dysfunction (29). In addition, a significant reduction of SCFA-producing bacteria, such as *Bacteroidetes* and *Clostridium*, led to a decrease in SCFAs production. The composition and structure of gut microbiota were properly regulated, which can contribute to the restoration of health (30). For instance, some herbal medicines changed the composition and structure of the gut microbial community to achieve anti-inflammatory and immunomodulatory effects, thereby contributing to alleviating ulcerative colitis (31, 32). Moreover, the compound polysaccharides containing yam and inulin polysaccharides ameliorated the experimental colitis of rats by decreasing the abundance of harmful bacteria and increasing the abundance of beneficial bacteria, such as SCFAs-producing bacteria lactic acid-producing bacteria, to reverse the dysregulated microbiota function (33).

Mice with AAD showing decrease diversity and abundance of intestinal flora and abnormal composition and functions of gut microbiota has been reported. Natural polysaccharides have shown a good effect in restoring the balance of the gut microbiota. *Panax ginseng* polysaccharides have been reported to significantly alter the composition and diversity of the gut microbiota by increasing the relative abundance of *Lactobacillus* and balanced metabolic processes by reversing carbohydrate, amino acid, and energy metabolism to normal levels (34). This research showed that CSP-1 regulated the gut microbial community of AAD mice by enriching *Phasecolarctobacterium, Bifidobacterium*, and reducing the abundance of *Parabacteroides, Sutterella, Coprobacillus* to near normal levels. Furthermore, CSP-1 also up-regulated the richness of intestinal flora. Probiotic bacteria in the intestinal flora have many beneficial effects on the host, which may greatly promote the recovery of mice with AAD. For instance, acetic acid and lactic acid which developed by the fermentation of *Bifidobacteria* can alter the acidity of the microbial ecosystem, thereby inhibiting the colonization of harmful intestinal flora (35). Furthermore, *Bifidobacteria* has also been reported to enhance immunity, resist pathogenic bacterial infection and exhibit anti-inflammatory activity (36). As SCFAs-producing bacteria, the increased abundance of *Phascolarctobacterium* exhibited positive effects on restoring the health of the host. The abundance of *Paracteroides* and *Coprobacillus* in MCL, MCM and MCH groups also returned to normal levels due to the effective regulation of CPS-1. Even for beneficial microorganisms, abnormal up-regulation of their relative abundance may not be beneficial to the host. *Sutterella wadsworthensis* reportedly may be a symbiotic, innocuous microbe in certain populations, such as those suffering from diarrhea-related intestinal diseases (20). The specific reasons for the reduced abundance of *Bacteroidetes* remained to be further studied.

It has been reported that the concentration of normal fecal anaerobic bacteria in mice with AAD was dramatically reduced due to the use of antibiotics, which leads to a decrease in carbohydrate metabolism and SCFAs (37). Fortunately, this research showed that three doses of CSP-1 promoted the production of SCFAs in AAD mice. This may be because CSP-1 is a prebiotic that cannot be digested by the stomach, and is successfully fermented by anaerobic microorganisms in the large intestine, thereby promoting the production of propionate butyrate acetate, etc. The increased production of SCFAs not only provides energy for intestinal cells helping maintain normal intestinal function, but also promotes intestinal water absorption improving dehydration in AAD mice (38). In addition, acetate and butyrate contributed to anti-inflammatory effects by activating GPR41 and GPR43 and inhibiting histone deacetylase (29). Thus, CSP-1 promoted the growth of SCFAs-associated bacteria, increased SCFAs production, inhibited inflammation, contributing to relieve antibiotic-related diarrhea in mice.

Antibiotics had a significant negative effect on the metabolism of amino acids in the body (39). It has been reported that the level of free amino acids in the stool may increase tenfold during diarrhea (40). LH inhibited the metabolism of amino acids in mice, which may lead to the accumulation of free amino acids. Fortunately, CSP-1 can significantly enhance the metabolism of amino acids in the body. Amino acids can enhance cell metabolism, increase protein synthesis by regulating protein translation, and increase mitochondrial content in skeletal muscle and adipocytes (41). The enhancement of amino acid metabolism by CSP-1 may also positively affect the translation process. Glycan biosynthesis and metabolism were closely related to many physiological and biochemical processes in the body, and its recovery was crucial for the maintenance of normal physiological homeostasis (42). Furthermore, CSP-1 also improved other metabolic functions of the gut microbiota in mice with AAD, involving digestive system, nervous system, translation, metabolic diseases, membrane transport, cell motility, transport and catabolism. Their mechanisms still need further studying.

**Figure 8:**
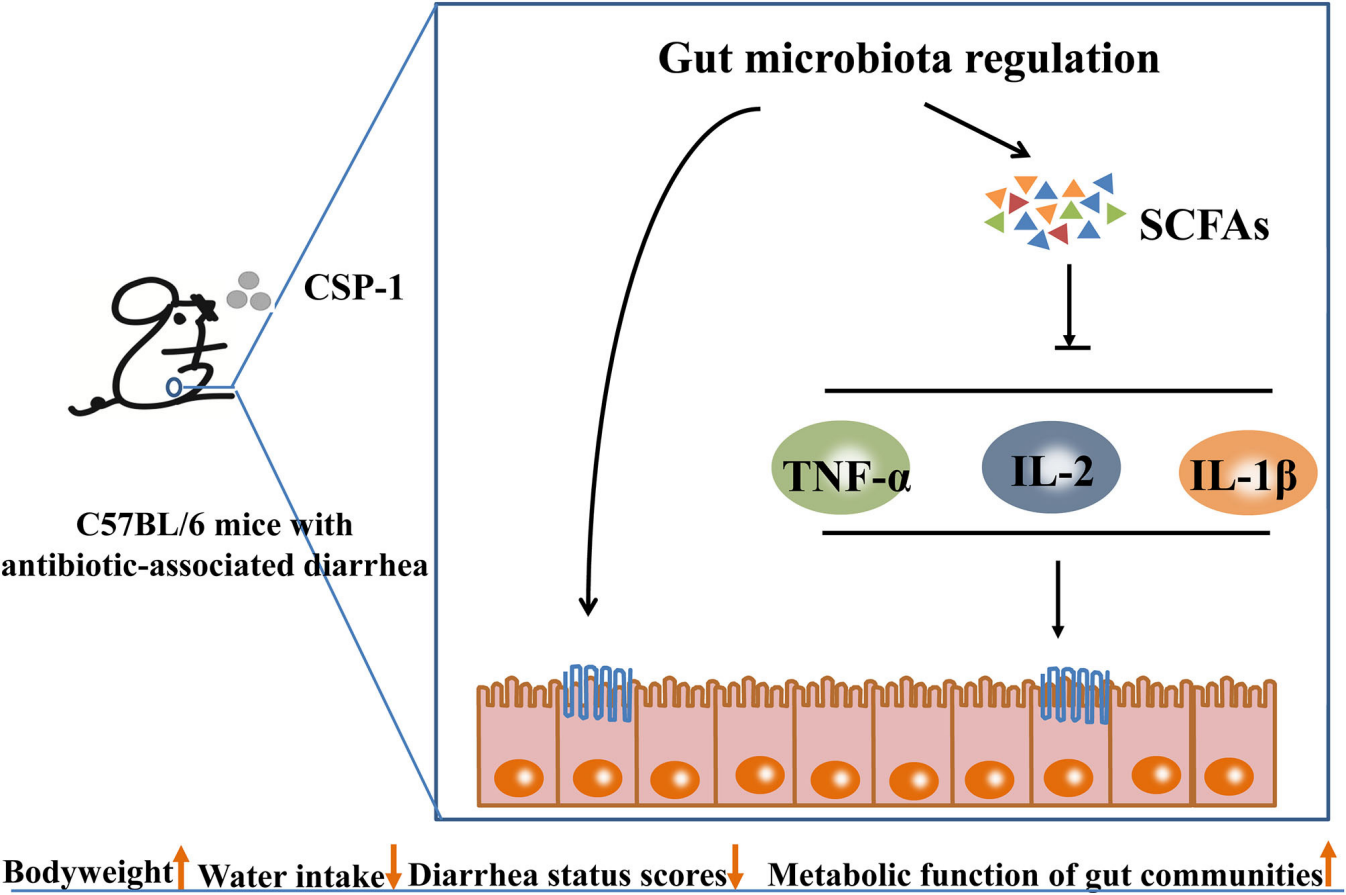
Schematic diagram of the mechanisms of CSP-1 alleviating AAD.

The schematic diagram of the mechanisms of CSP-1 alleviating AAD is shown in Figure 8. Overall, CSP-1 improved the intestinal barrier and regulated the gut microbiota and their metabolites as well as microbial metabolic function. On the basis of improving the composition and structure of intestinal flora, CSP-1 exhibited beneficial effects helping the recovery of AAD mice, which provided a basis for finding effective prebiotics for AAD.

## Data Availability Statement

The datasets presented in this study can be found in online repositories. The names of the repository/repositories and accession number(s) can be found at: https://www.ncbi.nlm.nih.gov/, SUB9203775.

## Ethics Statement

The animal study was reviewed and approved by Shanghai Ocean University.

## Author Contributions

KL and YJ conceived and designed research. MC, YW, and JW performed experiments. MC and YW analyzed data. JE contributed new reagents or analytical tools. MC wrote the manuscript. KL and JE revised the manuscript. All authors contributed to the article and approved the submitted version.

## Funding

The research was supported by the International Academic Cooperation of Science and Technology Committee of Shanghai, China (18430721100) and the National Natural Science Foundation of China (81572989).

## Conflict of Interest

The authors declare that the research was conducted in the absence of any commercial or financial relationships that could be construed as a potential conflict of interest.

## Publisher's Note

All claims expressed in this article are solely those of the authors and do not necessarily represent those of their affiliated organizations, or those of the publisher, the editors and the reviewers. Any product that may be evaluated in this article, or claim that may be made by its manufacturer, is not guaranteed or endorsed by the publisher.
