# Supplementary material for: Cereus sinensis Polysaccharide Alleviates Antibiotic-Associated Diarrhea Based on Modulating the Gut Microbiota in C57BL/6 Mice
Source: Front Nutr. 2021 Dec 13;8:751992. doi: 10.3389/fnut.2021.751992 (PMC8711652; doi:10.3389/fnut.2021.751992)
Supplement: Supplementary file 1 [file Image_1.pdf]

Supplementary Material

1 UV and FT-IR spectral analysis of CSP-1

The UV spectra of CSP-1 was shown in Figure S1a. There was weak absorbance at 280 nm in the UV spectrum of CSP-1, indicating the content of protein were very limited. The protein content of CSP-1 was 1.56% (1). The FT-IR spectra of CSP-1 was shown in Figure S1b. The strong band at 3268 cm^{-1} attributed to O-H stretching vibration. The band at 2933 cm^{-1} was assigned to C-H (CH, CH_2 and CH_3) stretching vibratio (2). The signals at 1637 cm^{-1} and 1545 cm^{-1} were characteristic absorption peaks of protein (3). The signals at 1451 cm^{-1} corresponded to the O-H bending vibration (4). The peaks at 1475-1300 cm^{-1} were the vibrations of C-H, which represented the deformations of CH2 groups (3). The band at 1243 cm^{-1} suggested the presence of S=O group (5, 6). The absorption peaks at 1000 cm^{-1} to 1200 cm^{-1} were C-O-C stretching vibration of pyranose and O-H bending vibration (7). The band at 831 cm^{-1} indicated the presence of α-glycosidic bonds in CSP-1.

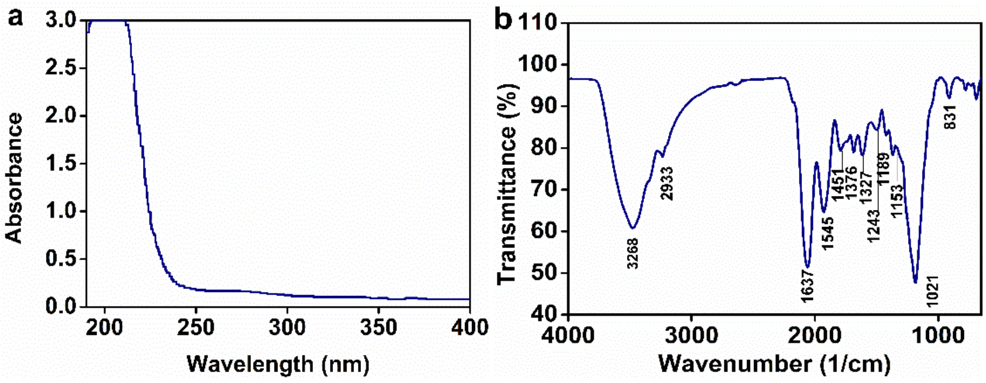

Fig. S1 UV spectra of CSP-1 (**a**) and FT-IR spectra of CSP-1 (**b**)

2 Reference

1. Cui M, Zhang M, Wu J, Han P, Lv M, Dong L, Liu K. Marine polysaccharides from Gelidium pacificum Okamura and Cereus sinensis reveal prebiotic functions. *Int J Biol Macromol.* (2020) 164:4381-4390. doi: 10.1016/j.ijbiomac.2020.08.255
2. Cui M, Wu J, Wang S, Shu H, Zhang M, Liu K, Liu K. Characterization and anti-inflammatory effects of sulfated polysaccharide from the red seaweed Gelidium pacificum Okamura. *Int J Biol Macromol*. (2019) 129:377-385. doi: 10.1016/j.ijbiomac.2019.02.043
3. Wang Y, Guo M. Purification and structural characterization of polysaccharides isolated from Auricularia cornea var. Li. *Carbohydr Polym*. (2020) 230:115680. doi: 10.1016/j.carbpol.2019.115680
4. Wang Y, Wei X, Wang F, Xu J, Tang X, Li N. Structural characterization and antioxidant activity of polysaccharide from ginger. *Int J Biol Macromol*. (2018) 111:862-869. doi: 10.1016/j.ijbiomac.2018.01.087
5. Seedevi P, Moovendhan M, Viramani S, Shanmugam A. Bioactive potential and structural chracterization of sulfated polysaccharide from seaweed (Gracilaria corticata). *Carbohydr*

Polym. (2017) 155:516-524. doi: 10.1016/j.carbpol.2016.09.011
6. Xia YG, Wang TL, Yu SM, Liang J, Kuang HX. Structural characteristics and hepatoprotective potential of Aralia elata root bark polysaccharides and their effects on SCFAs produced by intestinal flora metabolism. *Carbohydr Polym*. (2019) 207:256-265. doi: 10.1016/j.carbpol.2018.11.097
7. Li Y, Wang S, Sun Y, Xu W, Zheng H, Wang Y, et al. Apple polysaccharide protects ICR mice against colitis associated colorectal cancer through the regulation of microbial dysbiosis. *Carbohydr Polym*. (2020) 230:115726. doi: 10.1016/j.carbpol.2019.115726
